# Modeling and kinetic analysis of structural disintegration and biodegradation of biocomposite cultivating pots from agricultural waste

**DOI:** 10.1038/s41598-025-30302-z

**Published:** 2025-12-15

**Authors:** Manar E. Elashry, Elsayed G. Khater, Samir A. Ali

**Affiliations:** Agricultural and Biosystems Engineering Department, Faculty of Agriculture, Benha University, Banha, Egypt

**Keywords:** Biodegradation, Disintegration, Mineralization, Biocomposites, Cultivating pots, FTIR, PCA, Agricultural residues, Sugarcane bagasse, Lignocellulosic fillers, Mercerization pretreatment, Sustainable agriculture, Kinetic modeling,  palm wax

## Abstract

The growing global need for sustainable alternatives to synthetic plastics in agriculture has accelerated the development of biodegradable biocomposite cultivating pots derived from renewable agricultural residues. This study elucidates the biodegradation kinetics and structural disintegration mechanisms of cultivating pots formulated from palm wax, Lanette wax, and lignocellulosic fillers, including sugarcane bagasse, peat moss, compost, vermiculite, and activated carbon. The influence of mercerization pretreatment on degradation performance was systematically evaluated through disintegration assays, CO_2_ mineralization measurements, FTIR–ATR spectroscopy, and advanced kinetic modeling. After 90 days of composting, disintegration reached 64.18%, 66.70%, 67.20%, and 59.73% for P, PW, L, and LW pots, respectively, while carbon mineralization attained 65.98%, 70.53%, 70.08%, and 77.00%, indicating substantial biodegradation activity. Lanette wax-based composites containing pretreated fibers exhibited the most pronounced biodegradation response. Three kinetic models (Hill Sigmoid, Keursten, and soil respiration) were employed to describe the biodegradation behavior, among which the Hill Sigmoid model provided the best fit (R^2^ > 0.97), accurately capturing the non-linear kinetics of carbon release. FTIR spectral analysis confirmed progressive cleavage of C–O, C–H, and C=O bonds associated with cellulose, hemicellulose, and waxy matrices, evidencing microbial depolymerization. Principal Component Analysis (PCA) revealed that the carbon-to-nitrogen ratio and electrical conductivity were the most influential parameters governing biodegradation dynamics. Although the pots did not fully achieve the ISO 20200:2015 criterion of 90% disintegration within 90 days, their substantial degradation rates underscore strong potential for application in short-cycle crop cultivation. This study introduces a combined kinetic multivariate analytical framework for evaluating biocomposite degradation, offering predictive insights into compositional functional relationships. The findings advance the scientific basis for designing next-generation compostable pots, promoting soil health, waste valorization, and circular bioeconomy strategies in sustainable agriculture. Further optimization of filler composition and incorporation of bioactive additives is recommended to accelerate degradation and enhance regulatory compliance.

## Introduction

The widespread use of synthetic plastic containers in agriculture, particularly for seedling cultivation and nursery applications, poses growing environmental concerns due to their non-biodegradability and adverse effects on soil ecosystems. Although conventional plastic pots can be reused and are recyclable in principle, their practical reuse is limited by degradation and contamination during cultivation. Consequently, a considerable proportion ends up discarded, contributing to persistent plastic waste accumulation and potential soil contamination, disrupting microbial communities and reducing soil fertility. This issue is further amplified by increasing global agricultural intensification and the urgency to transition toward environmentally responsible and climate-smart farming practices^1^.

Biodegradable cultivating pots derived from renewable materials present a compelling alternative for sustainable crop production systems. Biocomposite pots, composed of natural binders and fibrous fillers from agricultural residues, are designed to degrade within the soil matrix, contributing to organic matter enrichment while eliminating plastic waste. Among these, palm wax and Lanette wax offer promising matrix systems due to their biodegradability and thermoplastic properties. Meanwhile, lignocellulosic fillers such as sugarcane bagasse, compost, peat moss, vermiculite, and activated carbon provide structural reinforcement and nutrient value. Pretreatment methods, such as mercerization with alkali, can enhance fiber-matrix adhesion and surface reactivity, thus accelerating microbial attack and breakdown.

The biodegradation of such materials is not only material-dependent but also influenced by environmental and physicochemical factors, including moisture content, carbon-to-nitrogen (C/N) ratio, and microbial activity. As previous studies suggest, the rhizosphere effect, the interaction between plant roots and soil microorganisms, may accelerate the decomposition of lignocellulosic components through enhanced enzymatic activity and nutrient solubilization^2,3^. However, achieving consistent and complete disintegration under standard composting conditions remains a challenge, especially for formulations that include hydrophobic or structurally complex components.

Despite growing interest, there remains a lack of comprehensive studies that integrate biodegradation performance with mechanistic insights into material breakdown. Most investigations focus on weight loss or surface-level assessments, without correlating biodegradation kinetics with molecular structural changes or validating models that predict degradation behavior.

Biodegradable cultivating pots composed of natural biocomposite materials provide one such alternative. These systems combine biodegradable matrices such as palm wax or Lanette wax with lignocellulosic fillers like sugarcane bagasse, compost, peat moss, vermiculite, and activated carbon to yield functional containers that can degrade in soil while returning organic content^4^, which demonstrated the structural viability and positive impact of the pots on plant growth. Such formulations aim to bridge structural performance and environmental compatibility, with pretreatments like mercerization (alkali treatment) enhancing fiber to matrix adhesion, reducing lignin/hemicellulose interference, and promoting microbial access.

However, the actual biodegradation behavior of these composites is governed by multiple interacting factors—not just material composition, but physicochemical conditions (moisture, temperature, C/N ratio), microbial dynamics, and structural characteristics. Prior studies often limit their scope to mass loss, morphological observation, or limited composting trials^5,6^. Comprehensive approaches that integrate degradation kinetics, molecular (e.g. FTIR) analysis, and multivariate statistical modeling remain sparse.

By coupling kinetic modeling, molecular insights, and multivariate analysis, this study aims to deliver predictive frameworks for designing compostable pot formulations. The results have direct relevance for sustainable agriculture, waste valorization, and reducing reliance on petrochemical plastics in horticultural systems.

The current research complements that study by addressing the environmental and soil-side performance through a detailed analysis of biodegradation kinetics, mineralization behavior, and structural disintegration mechanisms. By integrating the plant-side effects with degradation performance in composting conditions, the combined body of work offers a holistic understanding of the biocomposite pots’ full lifecycle impact. This progression underscores the importance of evaluating biodegradable materials not only in terms of plant compatibility but also in terms of their decomposition pathways and environmental integration within soil systems.

To address these gaps, this study presents a systematic evaluation of the biodegradation dynamics of biocomposite cultivating pots fabricated from palm and Lanette wax matrices combined with agricultural waste fillers. Emphasis is placed on the effect of mercerization pretreatment on biodegradability, as measured through disintegration rates, CO_2_ evolution (mineralization), and Fourier-transform infrared spectroscopy (FTIR) analysis. Furthermore, we employ and compare three mathematical models, including Hill sigmoid, Keursten, and soil respiration, to describe biodegradation kinetics. Principal component analysis (PCA) is also used to unravel the multivariate influence of material characteristics on biodegradation performance.

This integrated approach provides novel insights into optimizing biocomposite formulations for eco-friendly agricultural applications, with implications for sustainable resource management, waste valorization, and reduction of agricultural plastic dependency.

## Materials and methods

### Fabrication of biodegradable pots

Biodegradable cultivating pots were fabricated using palm wax (stearic acid) and Lanette wax (cetyl alcohol) as binding matrices at a wax ratio of 4:5 (wt%). Reinforcing fillers consisted of sugarcane bagasse, compost, peat moss, vermiculite, and activated carbon in proportions of 35 wt%, 35 wt%, 10 wt%, 10 wt%, and 10 wt%, respectively. All raw materials were locally sourced and processed to ensure uniform integration. The sugarcane bagasse was subjected to mercerization pretreatment using 1 M NaOH to enhance fiber–matrix adhesion by partially removing lignin and hemicellulose. The treated fibers were subsequently ground, washed, bleached, and oven-dried under controlled conditions to achieve homogeneity, as previously detailed in^4^.

The composite materials were melted-blended with a filler-to-binder weight ratio of 5:4, followed by compression molding at 160 bars and 60 °C. Mercerized and non-mercerized samples were fabricated, resulting in four categories:

- P-Pot: Palm wax-based, no pretreatment.
- PW-Pot: Palm wax-based, with pretreatment.
- L-Pot: Lanette wax-based, no pretreatment.
- LW-Pot: Lanette wax-based, with pretreatment.

### Carbon to nitrogen (C/N) ratio

CHNS (combustion) analysis is the accepted standard for accurately determining the bulk amounts of C, H, N, and S, and was our intended method for obtaining C/N ratios. Due to instrument unavailability at the time of analysis, we instead used SEM imaging with energy-dispersive X-ray spectroscopy (SEM–EDX) to obtain elemental maps and localized C and N counts on representative pot fragments. We emphasize that SEM–EDX is a surface-sensitive technique (with an information depth typically on the order of 1–2 µm) and its quantification of light elements, particularly N, is less reliable than combustion analysis. Therefore, EDX results were interpreted as relative/local compositional indicators rather than absolute bulk C/N values. To reduce sampling bias, we collected spectra from multiple, systematically selected regions (n = 3) per sample measuring (1 cm × 1 cm), including surface, cross-section, and filler-rich areas, and report averaged values ± SD. The observed spatial distributions of C and N were used to support mechanistic interpretations of the initial stages of biodegradation, which occur at and near the surface.

Previous studies in biochar and composite materials have used SEM–EDX/EDS to measure carbon and, where possible, oxygen/nitrogen contents locally and have demonstrated that such values correlate with bulk combustion analyses. For example, Ma et al. (2016)^7^ used multiple SEM–EDX measurement spots to map carbon content in biochar produced under varying pyrolysis temperatures, finding that maximum and average carbon content measured by SEM–EDX correlated well with total carbon from combustion methods. Similarly, Paula et al. (2025)^8^ used SEM/EDS to compare carbon‐rich composite formulations and interpret interfacial structure differences.

Considering such literature, the use of SEM–EDX in our study, although constrained by instrument availability, is supported insofar as it reveals trend differences among sample formulations and local compositional variations (surface vs cross section) that are relevant to biodegradation, even if absolute bulk C/N values remain approximate.

### FTIR spectroscopic analysis of the cultivating pots

FTIR spectra of the samples before and after composting revealed distinct changes in functional group intensities, confirming structural degradation. Key observations included the disappearance of OH and C≡C bands, indicating the breakdown of lignocellulosic and waxy compounds. Peaks associated with carboxylic acids (C=O stretching at ~ 1700 cm⁻^1^) and alkyl groups (C–H stretching at ~ 2920 cm⁻^1^) became more prominent post-degradation. The LW-pot exhibited the most significant spectral transformation, corroborating its superior biodegradability. The disappearance of fingerprint region peaks further indicated cleavage of polymer chains.

### Attenuated total reflectance fourier transform infrared (ATR-FTIR) spectra analysis of cultivating pots

Attenuated Total Reflectance Fourier Transform Infrared (ATR-FTIR) spectroscopy was applied to characterize the presence of functional groups within the matrices of the composite before and after ninety days of biodegradation. The spectra of each fabricated composite for the cultivating pots were acquired using the THERMO NICOLT iS50 FTIR Spectrometer, covering the range of 4000–500 cm^−1^^9^.

### Biodegradation properties for cultivating pots

**Fig. 1:**
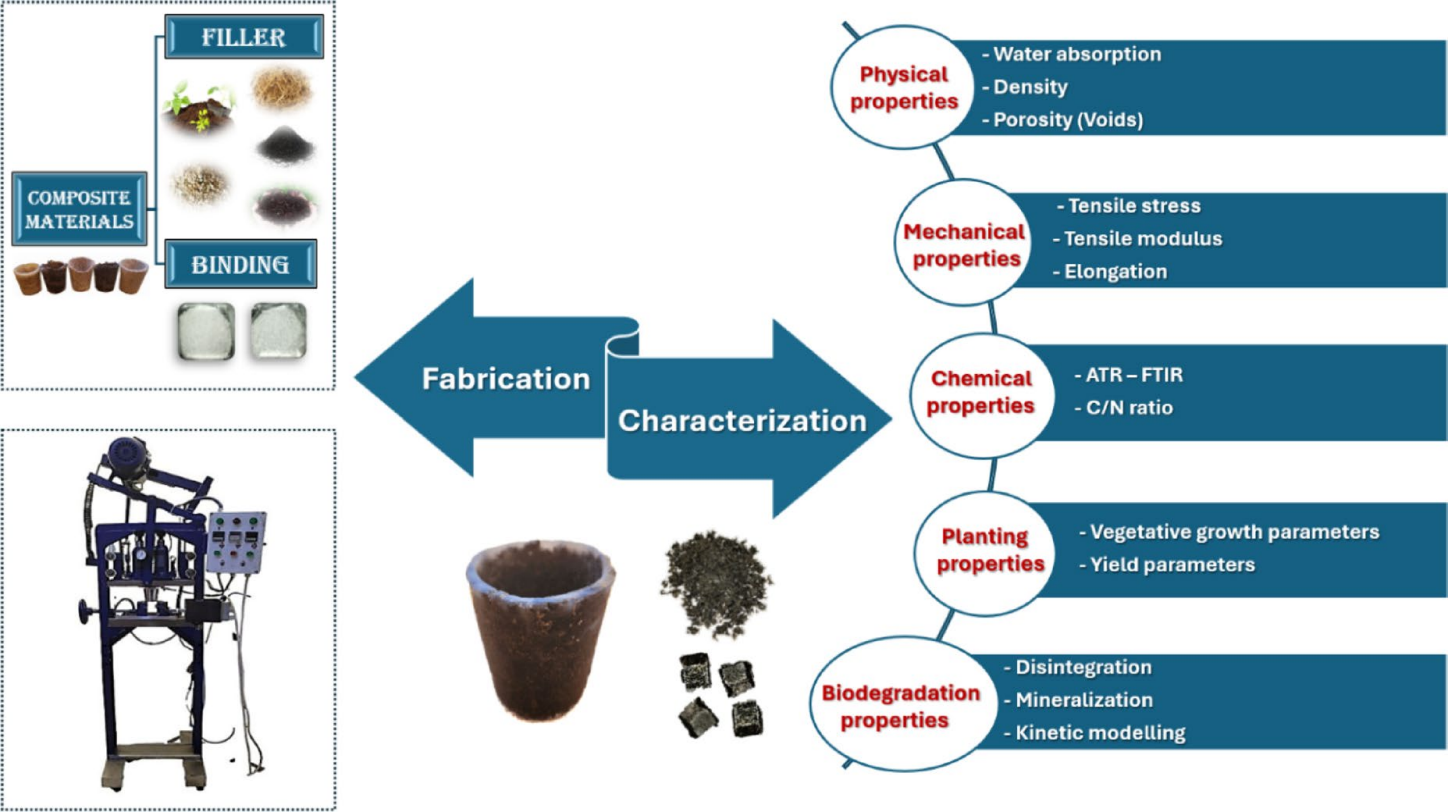
Fabrication and characterization parameters of biodegradable cultivating pots.

The biodegradability of cultivating pots was evaluated through different characteristics parameters, as illustrated in Fig. 1. Herein, the scope is the assessment of disintegration and the rate of carbon dioxide evolution over a period of 90 days.

### Disintegration of cultivating pots

The disintegration and biodegradation behavior of cultivating pot samples were evaluated under composting conditions adapted from ISO 20200:2015. A composting medium was prepared using a standardized mixture of soil, manure, and sand in a 2:1:1 ratio by weight. The specimens were buried at a depth of 10 cm within the compost under aerobic conditions and maintained at an ambient temperature. Samples were retrieved at five-day intervals over a designated testing period to assess visible disintegration and record weight loss. The percentage of weight loss was calculated to estimate the rate of biodegradation, as described in the methodology of^10^.

1 $${\text{W}}_{{\text{L}}} \% \, = \frac{Wi - Wt}{{Wi}} \times { 1}00\%$$

where W_L_ is denoted as the weight loss disintegration percentage of a sample, Wi is denoted as the initial weight of a sample, and Wt represents the weight of a sample after t days of compost burial.

### Biodegradation rate of cultivating pots

The degradation rate is estimated by measuring the CO_2_ evolution converted from the amount of carbon in the specimen. The percentage of biodegradation (D%) was calculated based on the kinetics of CO_2_ emissions evolved in each reactor^11^.

2 $${\text{D }}\% \, = \frac{{CO_{2, sample} - CO_{2, control} }}{{ThCO_{2} }}$$

where CO_2,sample_ is the total accumulated amount of CO_2_ sample from biodegradation, CO_2, control_ is the released amount of CO_2_ in a blank experiment, and ThCO_2_ is the theoretical or maximum liberated amount of CO_2_ from fully mineralization of the sample, calculated based on organic carbon content using the following equation^12^:

3 $${\text{ThCO}}_{{2}} = {\text{ C }} \times \, \left( {{44 } / { 12}} \right) \, \times {\text{ W}}$$

where C is the total organic carbon determined in the tested material (g), 44 is the molar mass of CO_2_, 12 is the atomic mass of carbon, and W is the sample weight in the bioreactor (g).

The quantification of CO_2_ during the biodegradation process employed the trapping and titration method, as described by^13^ This method is based on ISO 17556:2012 (Actual biodegradation in soil) with some adaptations from ISO 16929: 2013 (Disintegration in realistic composting setups). It involves capturing the CO_2_ emissions produced in the biodegradation process and assessing their concentration through a chemical titration process.

**Fig. 2:**
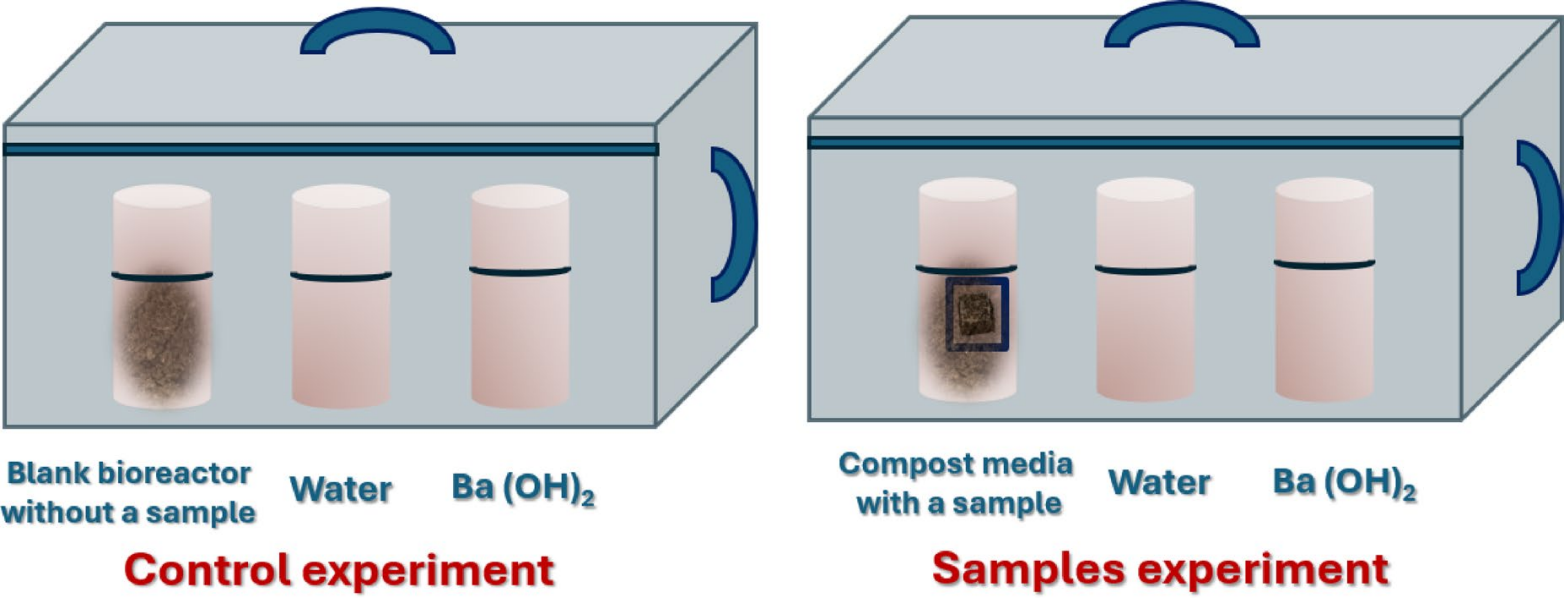
Mercerization experiment of pot samples.

The titration method includes trapping CO_2_ emissions using three plastic containers containing a burial compost sample as the disintegration media, water, and a dissolved amount of about 100 ml of 0.45 M barium hydroxide, Ba (OH)_2_ solution_,_ as a CO_2_ trap. The excess Ba (OH)_2_ in the solution is titrated with 0.1 M hydrochloric acid HCl using phenolphthalein as an indicator for the endpoint reaction. The system was refreshed every 5 days. The experimental setup (Fig. 2) consists of a large plastic box serving as a test bioreactor chamber containing the compost sample for CO_2_ quantification, and a blank bioreactor with a burial compost without a sample. The latter includes the CO_2_ capture solution, water for hydration, and the difference in CO_2_ levels between the two setups determines the amount of CO_2_ produced by the sample without the addition of the compost organisms^14^. All biodegradation experiments were conducted in triplicate bioreactors.

### Theoretical analytical models of the composites

#### Biodegradation kinetic models of the composites

Assess the cumulative carbon evolution rate and gain deeper insight through three mineralization kinetic models that were applied. These models include the Hill sigmoid model, the Keursten model^15^, and soil respiration models^16^, as represented by the following equations.

Hill sigmoid model:

4 $${\text{y }} = {\text{ y}}_{{{\text{max}}}} \times \frac{{T^{n} }}{{K^{n} + T^{n} }}$$

where y is the biodegradation rate at time (T) (days), y_max_ is the maximum biodegradation rate,

k and n are kinetic parameters of degradation.

Keursten model:

5 $${\text{y }} = {\text{ y}}_{{{\text{max}}}} \left[ {{1} e^{{\left( { - \alpha \left( {t - t_{lag} } \right)} \right)}} } \right]$$

where $\alpha$ is a degradation parameter.

The half-life time (T^1/2^) is obtained by:

6 $${\text{T}}^{{{1}/{2}}} \left( {{\text{days}}} \right) \, = {\text{ t}}_{{{\text{lag}}}} + \frac{ln2}{\alpha }$$

where T _lag_ is the time of the biodegradation lag phase.

Soil respiration model:

7 $${\text{y }} = {\text{ C}}\,t^{m}$$

C and m represent the initial mineralization rate.

Model fitting used the Generalized Reduced Gradient (GRG) Solver in Excel. Fit quality was assessed via χ^2^, coefficient of variation (C.V.), and RMSE.

#### Principal component analysis (PCA)

PCA was employed to assess multivariate relationships among variables such as carbon content, nitrogen, disintegration, mineralization, and tensile strength, enabling visualization of material class separation.

## Results and discussion

### Carbon to nitrogen (C/N) ratio

As illustrated in Table 1, it is reasonable to have palm wax-based pots (P and PW) with a high percentage of carbon at 54.4% and 49.3%, respectively, as they are formulated as (stearic acid, C_18_H_36_O_2_). On the other hand, the hexadecanol-based pots (L and LW), formulated as (cetyl alcohol, C_16_H_34_O), have carbon contents of 37% and 34.5%. It has been observed that both carbon and nitrogen decrease after pretreatment, and this decrement is due to the partial removal of hemicellulose and lignin. The C/N ratio of P, PW, L, and LW-pots was 4.77%, 4.79%, 5.3%, and 6.51%, respectively. However, the C/N ratio of L-pots was the highest, contrary to expectations, due to the nitrogen content effect on the ratio.

**Table 1:** Carbon to nitrogen ratios of biodegradable pots.

Label	Carbon (%)	Nitrogen (%)	Carbon to nitrogen (C/N)
P	54.4 ± 0.42	11.4 ± 0.35	4.77 ± 0.11
PW	49.3 ± 0.25	10.3 ± 0.25	4.79 ± 0.09
L	37 ± 1.05	5.8 ± 0.75	6.38 ± 0.67
LW	34.5 ± 0.45	5.3 ± 0.20	6.51 ± 0.16

As mentioned by^17^ the carbon-to-nitrogen (C/N) ratio has a key effect on the decomposition of biodegradable pots, with a greater value resulting in slower weight loss. Herein, the results contradict the findings of^17^, as discussed later in the subsequent disintegration results section, where the highest weight loss was found for the LW pot, inversely affected by the ratio. This discrepancy is due to his findings that had small or negligible nitrogen content values compared to the fabricated pots in this investigation, where nitrogen is an essential component for plant growth and soil enrichment.

### FTIR spectroscopic analysis of cultivating pots before and after biodegradation

**Fig. 3:**
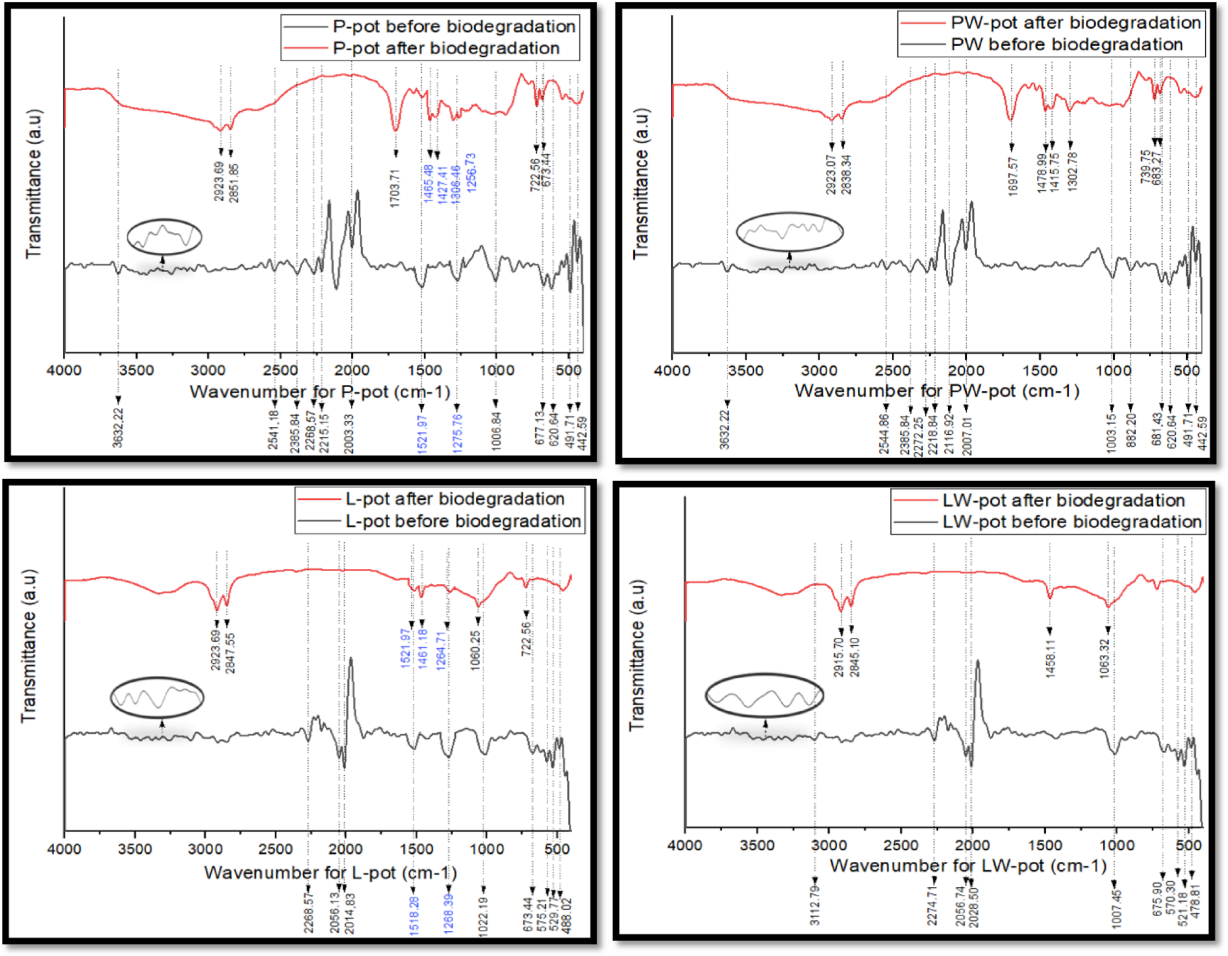
FTIR-ATR transmittance spectra of the cultivating pots before and after biodegradation.

Fourier Transform Infrared (FTIR) spectroscopy was employed to assess the structural and chemical transformations occurring in the biocomposite cultivating pots during the biodegradation process. Spectral analysis was conducted using Attenuated Total Reflectance (ATR) mode on all pot formulations (P, PW, L, LW) before and after 90 days of composting, as illustrated in Fig. 3. The results provide molecular-level insights into the degradation mechanisms and corroborate observations from disintegration and mineralization tests.

### Pre-biodegradation spectra

Each composite exhibited distinct spectral features reflective of its constituent materials. P-pots and PW-pots, containing palm wax and untreated or mercerized sugarcane bagasse, respectively, displayed broad OH stretching bands around 3500–3630 cm⁻^1^. These are attributed to hydroxyl groups from cellulose, hemicellulose, lignin, and residual organic matter originating from compost and peatmoss. High wavenumber peaks between 2540 and 2200 cm⁻^1^ were associated with activated carbon, compost residues, and vermiculite, including O–H stretch in carboxylic acids and possible CO_2_ absorption bands, and nitrile (C≡N) vibrations associated with the decomposition products in compost or the presence of carbonates in vermiculite. Peaks in the range of 1520–1275 cm⁻^1^ reflected C=C aromatic ring stretching of the aromatic guacyl (G) ring breathing as lignin rich in aromatic rings and C–O ester vibrations^18^, primarily from lignin and hemicellulose, or the bending properties of aromatic rings at the G and S lignin units appeared in the untreated sugarcane bagasse. Peaks in the fingerprint region at around 1006 cm^-1^ may be attributed to C–O stretching vibrations in fatty acids present in the palm wax or other oxygen-containing functional groups. Additionally, other remaining peaks may indicate C–H bending vibrations in alkanes, alkynes, or aromatic compounds, reflecting characteristic interactions or molecular structures of organic ingredients resulting from the combined components in the mixtures.

The L and LW pots, formulated with Lanette wax (cetyl alcohol), demonstrated additional peaks attributable to alkyne (C≡C) functional groups formed during reactions or interactions within the compounds, observed around 2050–2028 cm⁻^1^. The fingerprint region (1000–500 cm⁻^1^) revealed C–O and C–H bending vibrations associated with waxes, alcohols, and polysaccharides.

### Post-biodegradation spectra

Post-composting spectra displayed significant transformations in peak intensity and position. New medium-intensity peaks emerged at approximately 2920 and 2850 cm⁻^1^ in all samples, corresponding to C–H stretching in aliphatic chains, evidence of residual hydrocarbon structures that persisted after degradation. Hydrophobicity in the post-degraded samples does not originate from covalent bonding itself but rather from the presence of non-polar aliphatic and waxy residues. This hydrophobic nature can be mainly attributed to residual wax fractions, composed of long-chain hydrocarbons and esters, that resist microbial degradation.

The sharp OH stretching bands around 3632 cm⁻^1^ disappeared and other indistinguishable peaks before 2923.69 cm^-1^ in all formulations, confirming hydrolysis and microbial attack on hydroxyl-rich lignocellulosic components. This is consistent with previous studies^19,20^.

After composting, new medium-intensity peaks appeared near 2400–2000 cm⁻^1^, which are more likely associated with CO_2_ absorption from microbial respiration rather than triple-bond vibrations. Meanwhile, new bands appeared around 1700 cm⁻^1^, corresponding to C=O stretching of carboxylic acids formed during oxidative degradation. Shifts of lignin-related peaks (1520–1275 cm⁻^1^) toward 1470–1420 cm⁻^1^ indicated C–H deformation in methylene or methyl groups, signifying fragmentation of aromatic structures and formation of aliphatic residues.

It is plausible that these features persist or remain in the degraded compound, possibly resulting from anaerobic biodegradation or inherent structural characteristics of the molecule. The peaks are typically associated with the bending vibrations of methylene (–CH_2_–) groups, which constitute aliphatic chains in organic compounds, fragmentation of aromatic structures, and partial mineralization. New peaks at 1300–1250 cm^1^ represent C–O stretching, suggesting the form of low-energy ester linkages or syringyl ring units. As aligned with^21^. In the fingerprint region, the disappearance or attenuation of peaks, particularly at 1000 cm⁻^1^ and below, confirmed cleavage of ether and alkyl bonds, which typically arise when one hydrogen molecule from an alkane in the hydrocarbon is removed.

L- and LW-pots showed the most substantial structural collapse. LW-pot’s post-biodegradation spectra exhibited smoothed baselines, loss of high-energy triple bond peaks, and strong attenuation of all fingerprint peaks. Remaining bands around 1060 and 725 cm⁻^1^ in LW-pots represent residual aliphatic fragments or degradation intermediates. These spectral changes aligned closely with mineralization data and CO_2_ release, indicating near-complete structural disintegration.

Overall, the FTIR-ATR analysis confirmed the biochemical breakdown of both natural lignocellulosic components (bagasse, compost, activated carbon, and vermiculite) and semi-synthetic constituents (Lanette or Palm wax) within the composite matrix. Disappearance of OH, C≡C, and lignin-related peaks, along with the emergence of carboxylic acid and aliphatic signatures, corroborates the successful microbial degradation of the biocomposites. The extent of spectral transformation was most pronounced in LW-pots, consistent with their superior disintegration and CO_2_ evolution. These findings support the use of FTIR as a robust tool for monitoring biocomposite degradation and validate the material’s suitability for compostable agricultural applications.

All the abovementioned analyses align with the findings of^9,22–25^. A complete summary of spectral changes and corresponding functional group interpretations is presented in Table 2.

**Table 2:** Assignment and analysis of FTIR spectra for the cultivating pots prior and post degradation.

Peak No	Wavenumber (cm^-1^)								Chemical bond (functional group)	Chemical structure
	P-prior degradation	PW-prior degradation	L-prior degradation	LW-prior degradation	P-post degradation	PW-post degradation	L-post degradation	LW-post degradation		
1	3632.22	3632.22		3112.79					OH stretching	Vibrations from hydroxyl groups in sugarcane
2					2923.69 2851.85	2923.07 2838.34	2923.69 2847.55	2915.70 2845.10	C–H stretching	Vibrations in a covalent single bond are associated with carboxylic acid functional groups and alkyl groups, resulting from the hydrogen molecule of an alkane in the hydrocarbon
3	2541.18	2544.86							Unique functional groups (multiple bonds)	Bonding in activated carbon and the compost material’s structure, or highly strained molecular structures
4	2385.84	2385.84							O–H stretch	Specific bonding in the vermiculite or even in compost, possibly related to multiple bonds or specific mineral compositions such as O–H stretch in carboxylic acids
5	2268.57	2272.25	2268.57	2274.71					Carbon dioxide (CO_2_) or nitrile (C≡N) stretching	Vibrations are associated with the decomposition of products in compost or the presence of carbonates in vermiculite
6	2215.15	2218.84	2056.13	2056.74					(C≡C) stretching	Stretching of alkynes, formed during reactions
7	2003.33	2116.92 2007.01	2014.83	2028.50						
8					1703.71	1697.57			C=O stretching	Representing the disintegration to a low wavelength bond strength (double bonds) of the molecular structure of carboxylic acid functional groups
9	1521.97 1275.76		1518.28 1268.39				1521.97 1461.18 1264.71		C=C stretching or C–O stretching vibrations	C=C stretching of the aromatic guacyl (G) ring breathing as lignin rich in aromatic rings and C–O stretching vibrations present in ester functional groups within polysaccharides existing in lignin and hemicellulose or bending properties of aromatic rings at the G and S lignin units
10					1465.48 1427.41	1478.99 1415.75		1458.11	C–H deformation or (–CH_2_–) bending	- Deformation in the methylene or methyl group of the alkane compound or bending vibrations of methylene groups constitute aliphatic chains in the molecular plane of organic compounds - Scissoring vibrations due to alterations in molecular rearrangements post-biodegradation - Motion of carbon-hydrogen bonds, commonly found in alkane or alkyl groups, depending on the type of C–H bond (methyl, methylene, or methine), resulting from anaerobic biodegradation and the molecule’s structure
11	1006.84	1003.15	1022.19	1007.45	1306.46 1256.73		1060.25	1063.32	C–O stretching	- Vibrations in fatty acids are present in palm wax or other oxygen-containing functional groups - Stretching in aromatic ester and syringyl ring - Stretching vibrations in ethers, possibly originating from the cetyl alcohol
12	677.13 620.64 491.71 442.59	882.20 681.43 620.64 491.71 442.59	673.44 575.21 529.77 488.02	675.90 570.30 521.18 478.81	722.56 673.44	739.75 683.27	722.56	725.63 457.94	C–H bending	Vibrations in alkanes, alkynes, or aromatic compounds are attributed to characteristic interactions

### Biodegradation of cultivating pots

#### Disintegration performance of the cultivating pots

**Fig. 4:**
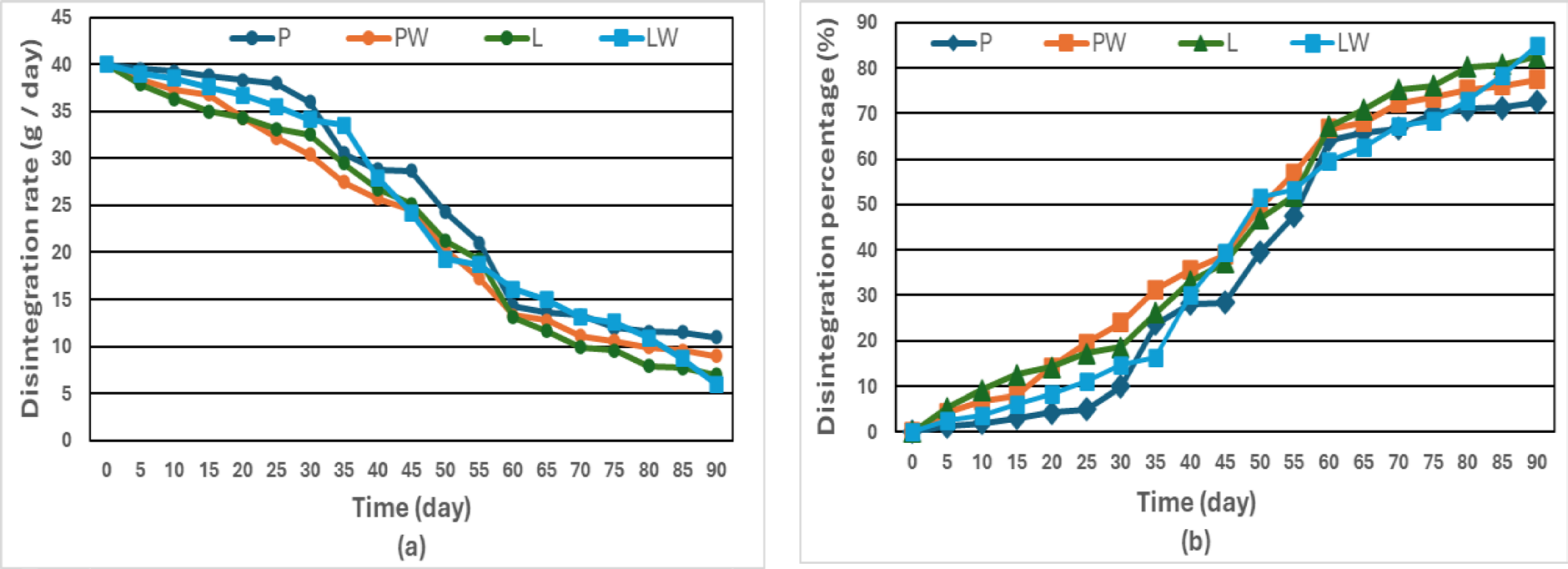
**a** Disintegration rate and **b** Percentage of disintegration of the cultivating pots.

A 40 g piece of each composted cultivating pot was monitored over a 90-day composting period with measurements taken every five days to assess weight loss resulting from biodegradation through statistical ANOVA (Table 3). Results showed a progressive increase in weight disintegration, indicating effective microbial degradation. Figure 4 depicts the disintegration rate and percentage of disintegration, respectively.

**Table 3:** Analysis of variance for disintegration rate.

Source	DF	Adj SS	Adj MS	F-value	*P*-value
Type	3	304.8	101.6	0.818	0.485
Error	212	26,344.5	124.3		
Total	215	26,649.3

**Fig. 5:**
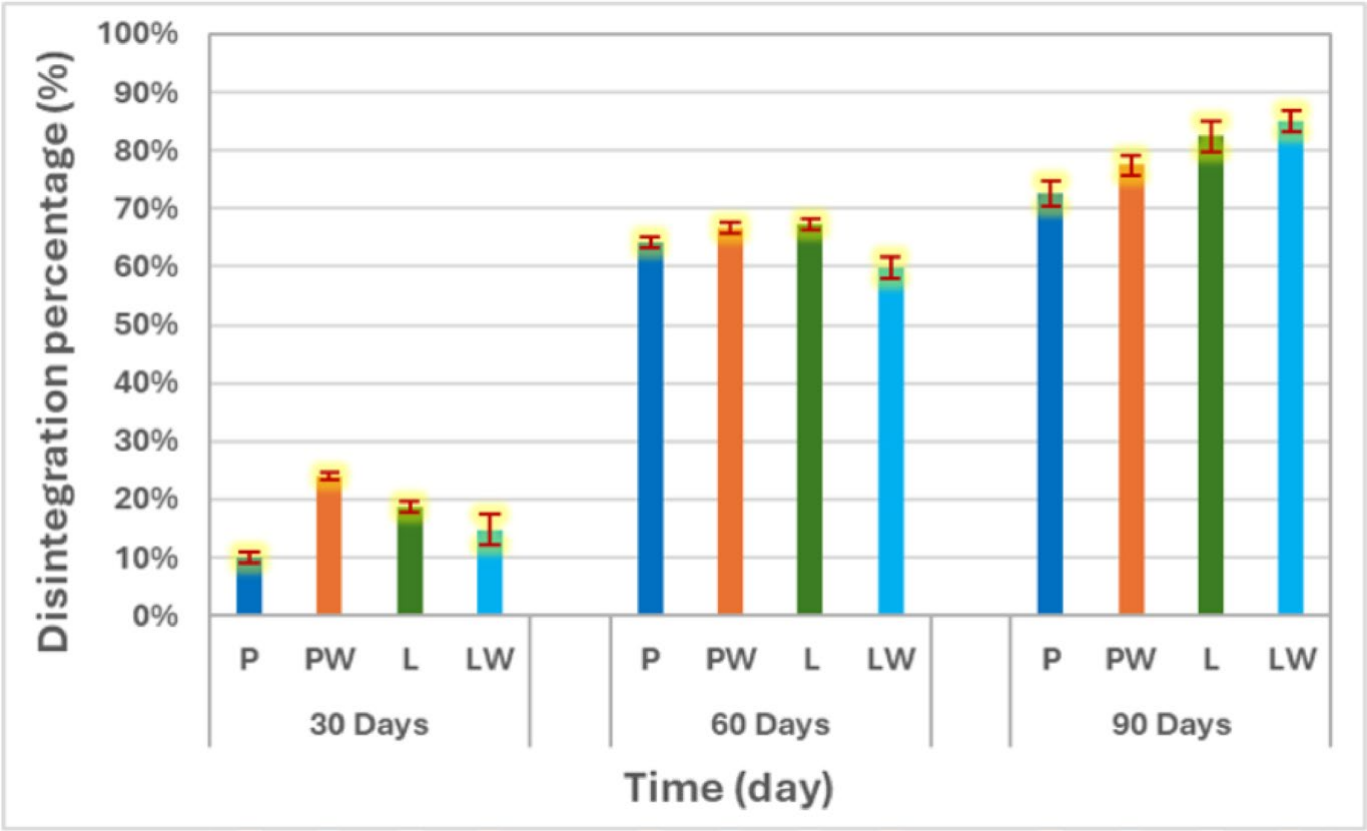
Comparisons of the disintegration percentages for each cultivating pot after each month.

The summarized percentages of disintegration, as presented in Fig. 5, for P, PW, L, and LW after 30 days were 10, 24, 18.65, and 14.73%, respectively. After 60 days, these values increased to 64.18, 66.70, 67.20, and 59.73%, and after 90 days, they reached 72.50, 77.50, 82.50, and 85%. Among the tested treatments, LW showed a comparatively higher disintegration trend, followed by L, PW, and P in descending order. The LW pot consistently outperformed others, demonstrating the synergistic effect of Lanette wax and mercerized fillers on biodegradability. The results also confirmed the positive influence of alkali pretreatment on enhancing filler-matrix compatibility and facilitating microbial access. Mercerization pretreatment contributed to improved fiber-matrix interfacial bonding and increased surface roughness, enhancing microbial adhesion and accelerating hydrolysis of hemicellulosic components. The increased wettability and removal of amorphous lignin fractions promote enzymatic access to cellulose chains, leading to faster fragmentation of the composite structure.

**Fig. 6:**
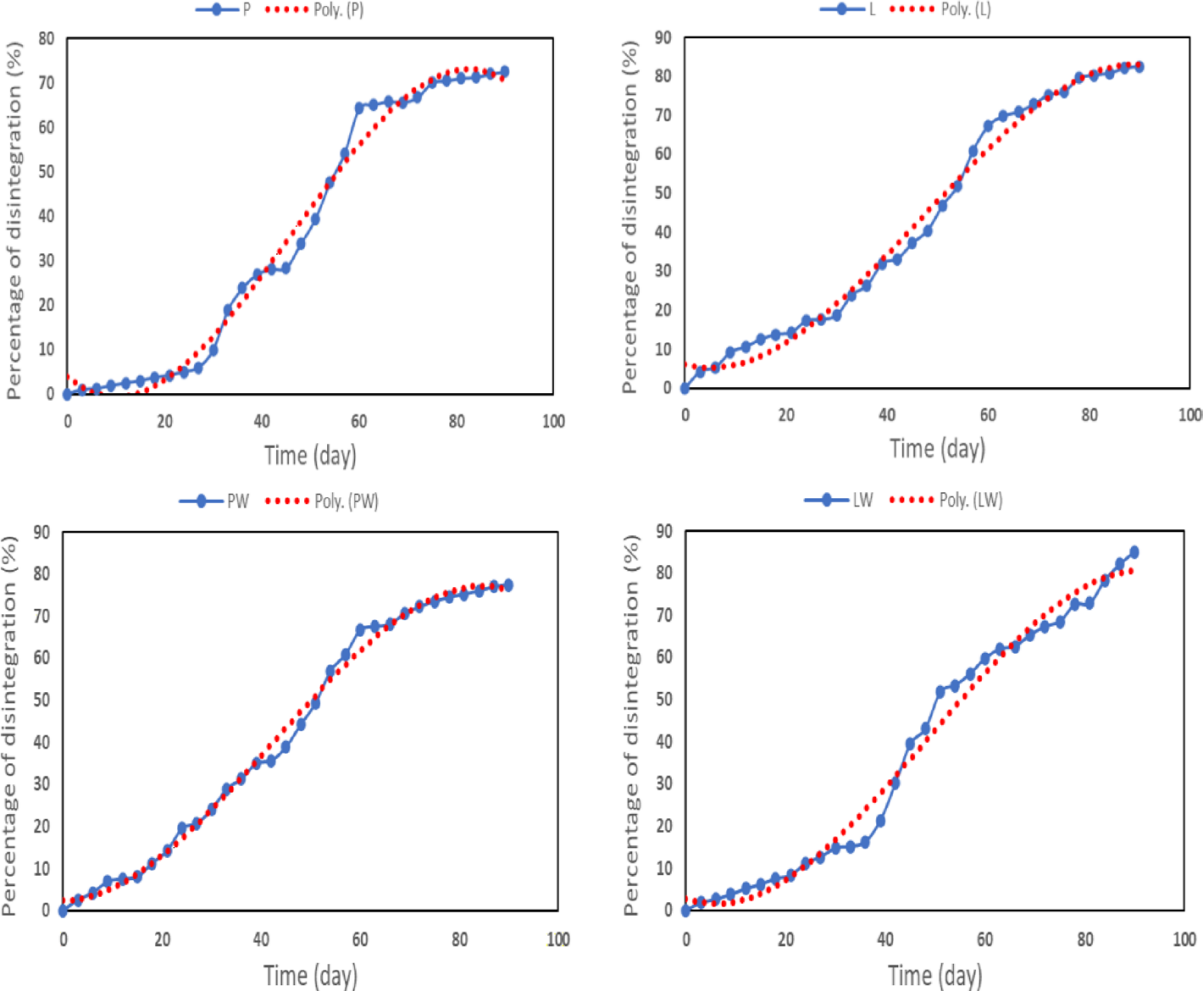
Regression analysis of the disintegration percentage for various types of cultivating pots.

As depicted in Fig. 6, regression analysis was conducted to determine the governing equation for the biodegradation of different types of cultivating pots.

The disintegration data yielded strong polynomial fits (R^2^ > 0.98), supporting the reliability of the degradation trends observed. The equation formula is as follows:

8 $${\text{Disintegration }}\left( {\text{D}} \right) \, \left( \% \right) \, = {\text{ a }}\,{\text{T}}^{{3}} + {\text{ b}}\,{\text{ T}}^{{2}} + {\text{ c}}\,{\text{ T }} + {\text{ d}}$$

where T is denoted as the time in days, a, b, c, and d, are equation constants, depending on the cultivating pot types as calculated in the following Table 4.

**Table 4:** Constants of the disintegration equation for different cultivating pot types.

Label	a ± SE	b ± SE	c ± SE	d ± SE	R^2^
P	− 0.0004 ± 0.2728*	0.0534 ± 0.0072*	-0.0561 ± 0.0001*	4.0244 ± 2.7585	0.9885
PW	− 0.0002 ± 0.1593	0.0307 ± 0.0042*	0.0204 ± 0.00003*	2.3282 ± 1.6103	0.995
L	− 0.0003 ± 0.2508	0.0367 ± 0.0066*	0.3506 ± 0.00005*	6.1467 ± 2.5355	0.9894
LW	− 0.0003 ± 0.2914	0.0371 ± 0.0076*	0.4199 ± 0.0001*	2.8071 ± 2.9460	0.9852

Although some coefficients have relatively high p-values, all constants and their standard errors are presented here for transparency. The overall model fit (R^2^ > 0.98) indicates that the polynomial equations reliably describe the degradation trends.

It should be noted that regulatory standards prescribe a requirement for the disintegration of biodegradable materials to be at least 90% within 90 days in anaerobic conditions, as stipulated by^26^. Despite high degradation rates, unfortunately, none of the formulations met the 90% disintegration threshold required by ISO 20,200 within 90 days, suggesting the need for further optimization through reducing wax content, improving wax dispersion, adding bioactive additives, or blending with more labile polymers, strategies that we propose for future work.

The biodegradation and disintegration of pot materials over 90 days depend strongly on polymer chemistry, composite formulation (filler content, coatings), and the composting regime (industrial/thermophilic vs. home/ambient). Under industrial composting (thermophilic, ≈ 55–58 °C), PLA powders and some PLA products can show high biodegradation or disintegration (e.g., ≈88% biodegradation/near-complete disintegration in pilot-scale tests)^27^. Polyhydroxyalkanoates (PHAs, e.g., PHB/PHBV) are among the most rapidly biodegraded polyesters under standard composting tests, and multiple studies report ≥ 90% physical disintegration or high biodegradation within 90 days under thermophilic conditions, although additives and lignocellulosic fillers can change the rate^28,29^. Thermoplastic starch (TPS) composites and TPS/PCL blends display wide variability; some TPS-rich biocomposites lose only ~ 30–40% mass after 90 days under realistic compost or pilot-scale conditions (with large reductions in mechanical strength), particularly when hydrophobic phases or low water uptake limit hydrolysis^30,31^. Finally, comparative tests of commercial “compostable” products show wide real-world variability: some certified items do not reach 90% disintegration in 90 days in laboratory-scale industrial composting tests, highlighting the influence of product form and testing setup^32^.

Despite the hydrophobic nature of waxes, the incorporation of lignocellulosic fibers and compost enhanced microbial colonization and oxygen transfer, leading to degradation rates comparable to or exceeding several commercial biodegradable systems such as PLA or PHBV composites.

#### Mineralization of cultivating pots

A 40 g piece of each composted cultivating pot was analyzed for CO_2_ evolution every five days over three months to assess mineralization resulting from biodegradation. CO_2_ evolution rates mirrored the disintegration behavior, indicating parallel mineralization activity.

**Fig. 7:**
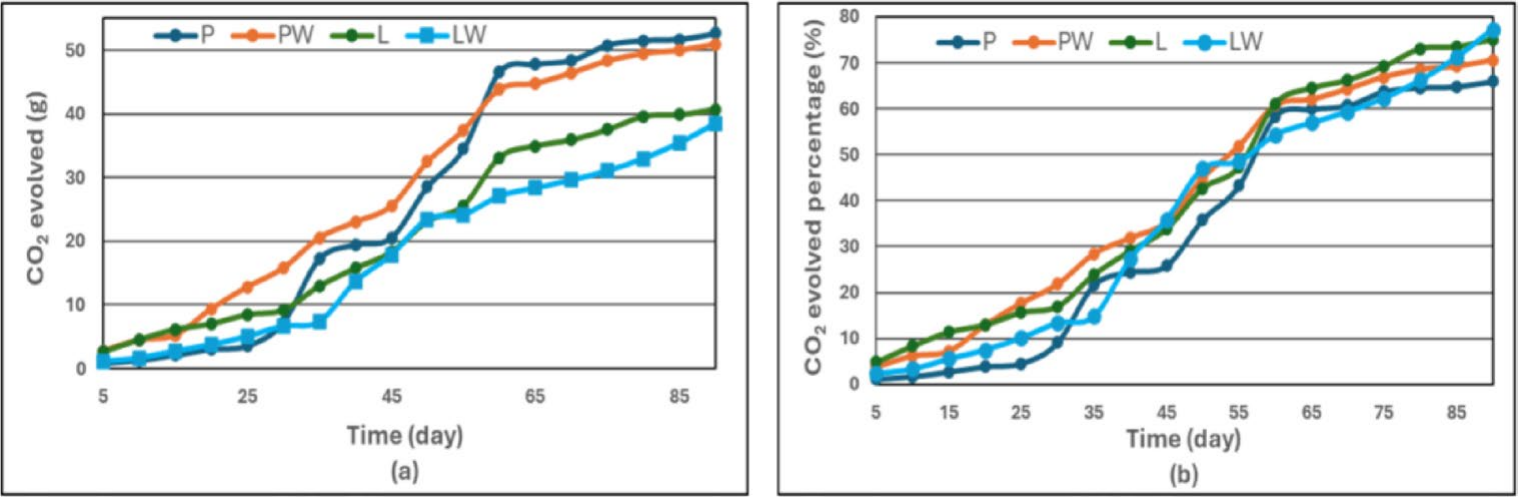
**a** CO_2_ evolution rate and **b** CO_2_ evolved percentage of the cultivating pots.

**Fig. 8:**
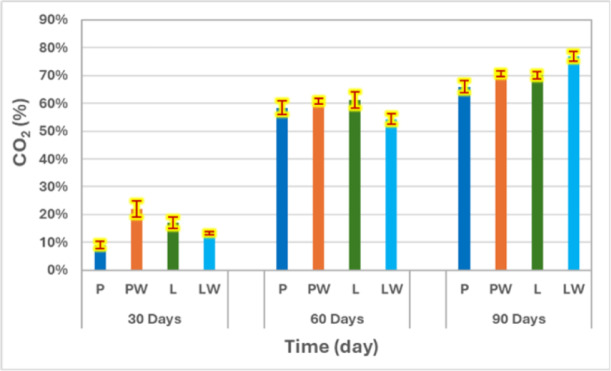
Comparisons of the CO_2_ evolved percentages for each cultivating pot after each month.

Figure 7 illustrates the temporal CO_2_ evolution rate and its corresponding percentage over the 90-day incubation period, whereas Fig. 8 presents the cumulative CO_2_ evolved (mineralization percentage) using a statistical ANOVA (Table 5). The LW treatment showed a lower CO_2_ evolution rate at the final stage (day 90), indicating that most of its biodegradation activity had already occurred earlier in the incubation. Consequently, LW accumulated the highest total CO_2_ evolution by day 90 (Fig. 7), reflecting its greater overall mineralization extent compared with the other treatments (LW > L > PW > P).

**Table 5:** Analysis of variance for CO_2_ evolution rate.

Source	DF	Adj SS	Adj MS	F-value	*P*-value
Type	3	3878	1292.6	4.832	0.003
Error	212	56,712	267.5		
Total	215	60,590			

The slower mineralization observed for palm wax-based pots (P, PW) can be attributed to the higher long-chain fatty acid crystallinity, which resists microbial attack and retards oxygen diffusion. Lanette wax, in contrast, contains hydroxylated aliphatic chains that are more susceptible to oxidation, explaining the enhanced biodegradation kinetics observed.

The summarized percentages of CO_2_ evolved, as presented in Fig. 8, for P, PW, L, and LW after 30 days were 9%, 22%, 16.97%, and 13.40%, respectively. After 60 days, these values increased to 58.40%, 60.70%, 61.15%, and 54.35%, and after 90 days, they reached 65.98%, 70.53%, 70.08%, and 77%. Among the tested treatments, LW showed a comparatively higher mineralization rate, followed by L, PW, and P in descending order. Mercerization pretreatment was observed to enhance biodegradation properties, reducing the time required for degradation. The CO_2_ measurements confirmed the composite’s microbial biodegradability under aerobic composting conditions. These findings were consistent with previous studies on biocomposites^9,19^.

#### Kinetic models of biodegradation for cultivating pots

**Fig. 9:**
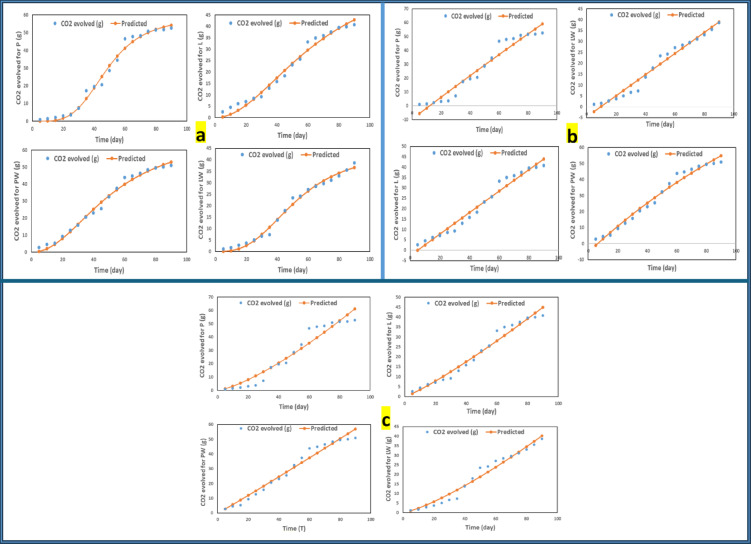
**a** Hill sigmoid, **b** Keursten, and **c** Soil respiration modeling curve of CO_2_ evolved for cultivating pots.

A Generalized Reduced Gradient (GRG) nonlinear sum least square residuals (SSR) data fitting was performed on the three models using Excel Solver, as illustrated in Fig. 9, to gain a deeper insight into the carbon dioxide evolution rate of the biodegradable cultivating pots. The parameters for the Hill sigmoid, Keursten, and soil respiration models are compared based on χ^2^, C.V., and RMSE in Table 6, which is provided in Table 7.

**Table 6:** Comparisons among fitting models for various types of cultivating pots using different indices.

Label	Hill sigmoid model			Keursten model			Soil respiration model		
	χ^2^	C.V	RMSE	χ^2^	C.V	RMSE	χ^2^	C.V	RMSE
P	2.38E-20	1.0870	2.4043	0.9999	1.1530	4.7489	0.1600	1.1525	5.1777
PW	0.536175	1.1278	1.8683	–	1.15687	2.6768	0.9903	1.1521	3.0846
L	0.092208	1.1272	1.9658	–	1.15292	2.4237	0.9972	1.1509	2.3669
LW	0.014078	1.1070	1.4174	0.8813	1.15291	2.2357	0.9783	1.1520	2.3405

*Note* Missing χ^2^ values for PW and L in the Keursten model indicate non-convergent fitting or undefined residual variance, likely associated with the early lag phase of microbial depolymerization, as shown in Fig. 9b.

**Table 7:** Hill sigmoid, Keursten, and Soil respiration fitting equation constants.

Label	Hill sigmoid model				Keursten model					Soil respiration model		
	y_max_	k	n	R^2^	y_max_	α	t_lag_ = T_1/2_ − (ln2/α)	T1/2	R^2^	C	m	R^2^
P	58.90	48.36	3.97	0.97	765.75	0.001	12.16	22.26	0.999	0.142	1.348	0.99
PW	69.97	52.47	2.11	0.99	141.50	0.006	6.28	22.37	0.995	0.541	1.035	0.99
L	67.01	67.50	1.99	0.99	2512.15	0.000	5.19	22.37	0.999	0.240	1.163	0.99
LW	44.47	52.61	2.86	0.98	4955.80	0.000	9.56	22.37	0.999	0.119	1.294	0.99

The most optimal model, identified through the lowest comparison indexes (χ^2^, C.V, and RMSE), was the Hill Sigmoid model. The inclusion of parameter standard errors and confidence intervals further supports the robustness of this model, confirming that the fitted parameters were statistically significant (*p* < 0.05). The sigmoidal degradation profile corresponds to the composite’s cellulose-based matrix, consistent with previous findings^14^. The palm wax component in the composite serves as a hydrophobic water repellent agent, not impacting t_lag_ in the Kuersten model designed for water diffusivity^15^. Regarding the soil respiration model, it corresponds to a first-order kinetic process^16^, herein, the weight loss due to biodegradation fits a polynomial function.

In the Keursten model, the calculation of χ^2^ for the PW and L treatments was impractical, as the fitting displayed negative parameter regions (approximately − 1 to − 10), reflecting the early lag phase^14^ of microbial depolymerization and macromolecular chain cleavage by extracellular enzymes (Fig. 9b). This initial nonlinearity likely hindered proper model convergence and, consequently, prevented the determination of χ^2^ for these datasets. Moreover, the calculation of χ^2^ for the PW and L treatments in the Keursten model was impractical because the fitting did not converge within a meaningful parameter range. This behavior, as illustrated in Fig. 9b, corresponds to the early lag phase of microbial depolymerization and chain cleavage, which produced low residual variance and nonlinear trends during the initial stage, thereby preventing a reliable estimation of χ^2^.

**Fig. 10:**
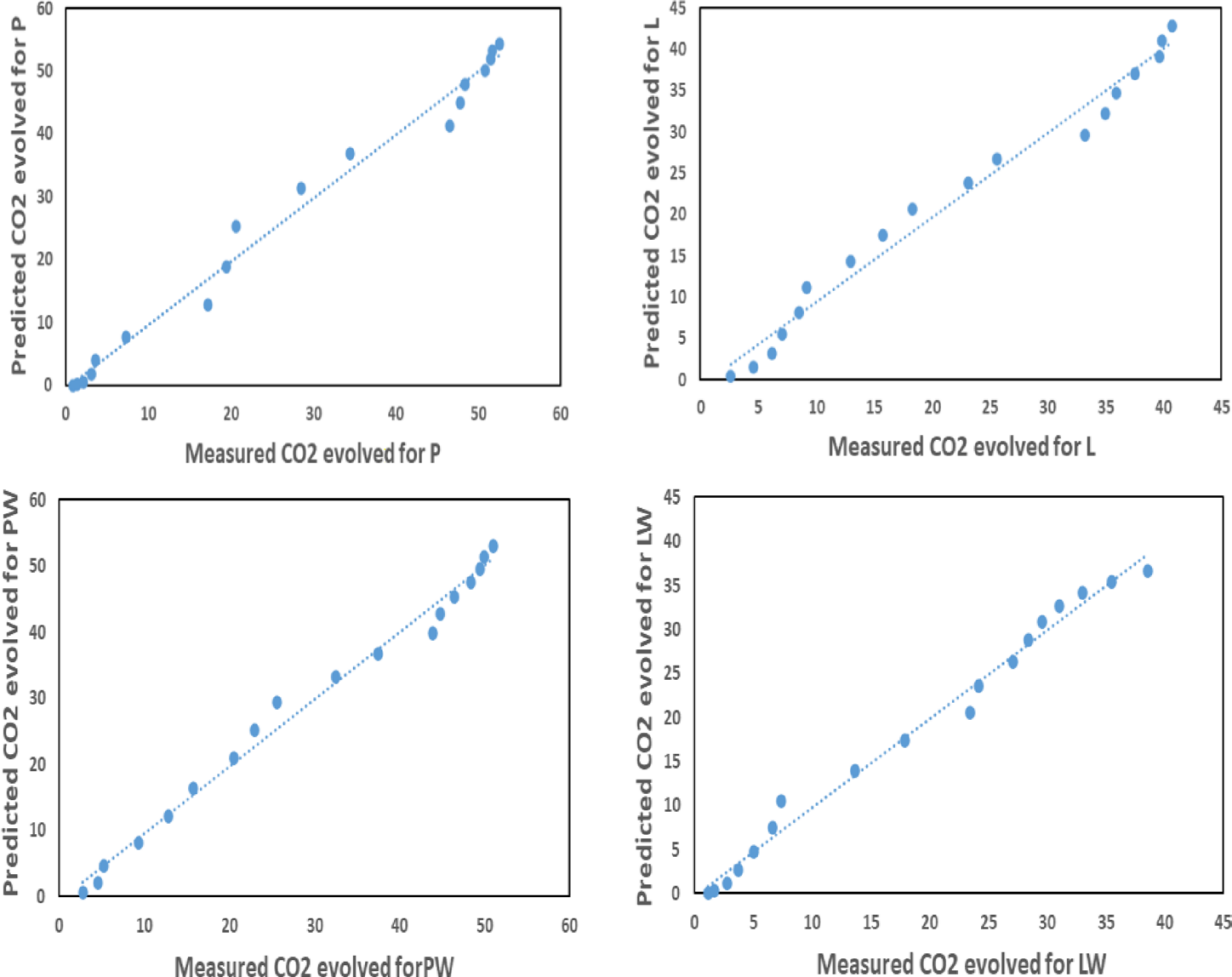
Comparative analysis of predicted CO_2_ evolution across diverse cultivating pots in contrast to the measured CO_2_ evolution.

To validate the predicted values of the best-fitting model, "Hill Sigmoid," against actual observations, the predicted biodegradation rates were graphically represented against the measured rates, as depicted in Fig. 10, and expressed by the following equation:

9 $${\text{CO}}_{{2}} \,{\text{predicted }} = {\text{ A }}\,{\text{CO}}_{{2}} \,{\text{measured }} + {\text{ B}}$$

where A and B are the constants of the fitting equation for validation, as calculated in Table 8.

**Table 8:** Validation fitting equation parameters.

Label	A	B	R^2^ (%)
P	1.0062	− 0.4764	98.66
PW	1.0156	− 0.7348	98.93
L	1.0218	− 0.8407	98.16
LW	1.0104	− 0.3937	98.84

#### Multivariate principal component descriptive analysis (2D-PCA)

PCA provides a multidimensional correlation approach for multi-response differentiation and optimization^33^, This reveals how variables such as tensile strength, electrical conductivity (EC), and the C/N ratio collectively influence biodegradation behavior across the different formulations. This dimensional reduction approach clarifies how each treatment (e.g., LW, L, PW, and P) forms a distinct cluster according to its specific physicochemical and mechanical characteristics, thereby confirming the interplay between structural modification and biodegradability. The application of PCA in this context follows recent practices in materials and biodegradation research, where it is widely used to uncover correlated responses and dependencies that are not evident from univariate analyses.

**Fig. 11:**
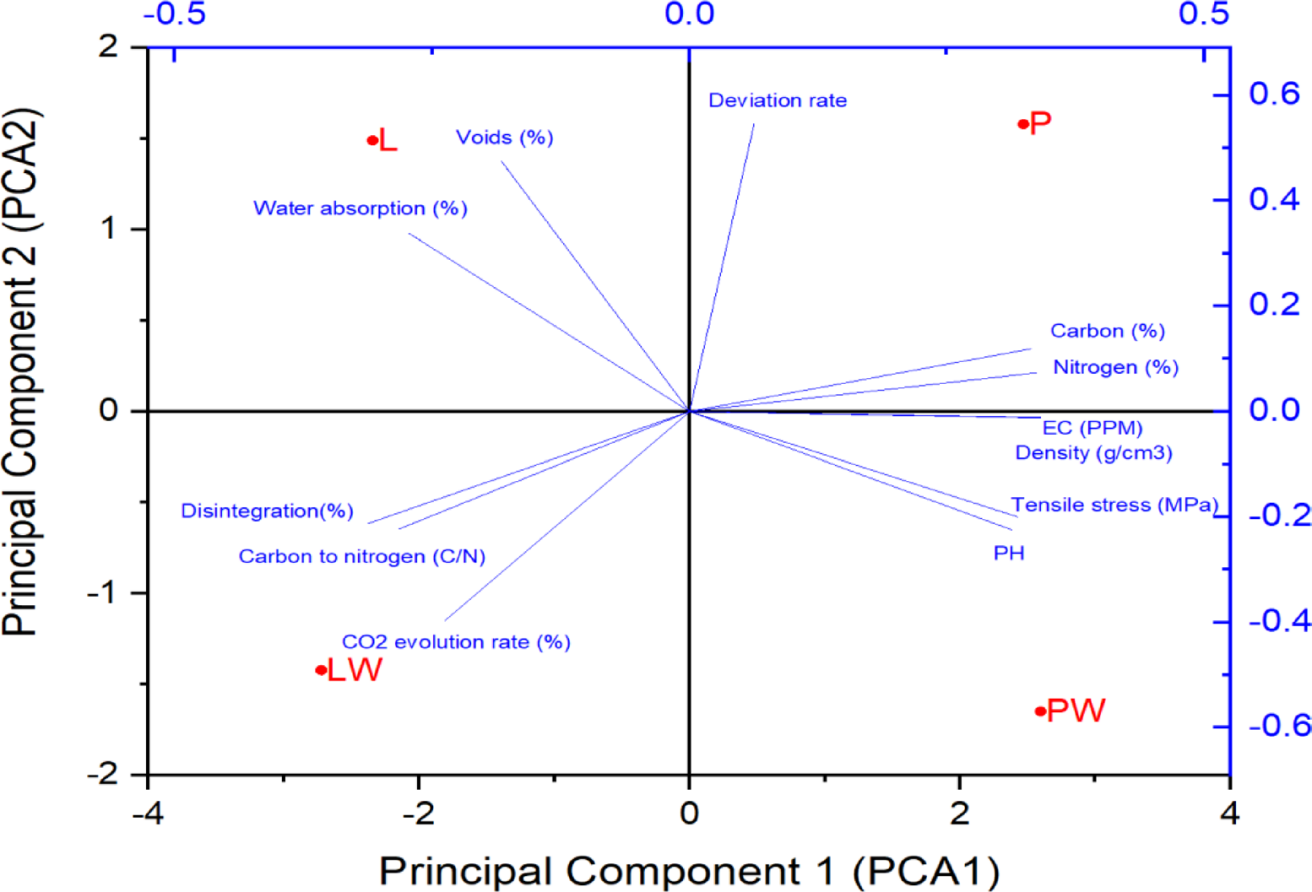
2D-PCA of the variable sets for cultivating pots.

In a summary of the aforementioned attributes and their interrelationships, as graphically represented in Fig. 11, to elucidate the linear combination of variables. Notably, the P-pot aligns with PCA2, influenced by deviation rate, carbon, nitrogen, and EC, whereas the PW-pot conforms to PCA1, driven by density, tensile stress, and pH.

The application of 2D-PCA reveals a stratification of the fabricated materials into four discernible classes, affirming the transformative impact of mercerization pretreatment in instilling novel characteristics within composites for pots derived from identical source materials.

As previously discussed, the C/N ratio exhibits a correlation with biodegradability properties, influencing both disintegration and the rate of carbon dioxide evolution. This relationship manifests in enhanced biodegradation in LW-pots. Intriguingly, nitrogen inversely correlates with the C/N ratio, implying an inverse relationship with biodegradability properties. Conversely, while carbon exhibits a direct correlation with the C/N ratio, it surprisingly shows an inverse correlation with biodegradability. This paradox is rationalized by the heavier carbon weight necessitating prolonged biodegradation time. Concurrently, elevated electrical conductivity (EC) signifies a higher concentration of elements impacting the biodegradation paradigm.

Further logical associations emerge, where water absorption and voids inversely correlate with density and tensile properties. Increased voids contribute to heightened water absorption, culminating in reduced density and subsequently diminished tensile stress.

## Conclusion

This study comprehensively explored the biodegradation kinetics and structural disintegration behavior of biocomposite cultivating pots fabricated from agricultural residues, emphasizing the influence of mercerization pretreatment on enhancing degradation dynamics. The integration of kinetic modeling and principal component analysis (PCA) provided a multidimensional understanding of how compositional factors, pretreatment, and physicochemical attributes govern biodegradation performance. Among the three applied kinetic models—Hill Sigmoid, Keursten, and soil respiration—the Hill Sigmoid model exhibited the most accurate fit, as evidenced by the lowest χ^2^, C.V., and RMSE values, reflecting its superior ability to describe the degradation kinetics of cellulose-rich biocomposites. Regression analysis further elucidated the governing mathematical relationships between biodegradation rates and compositional parameters across pot formulations.

The mercerization pretreatment significantly improved the biodegradation potential by partially removing hemicellulose and lignin, thereby enhancing fiber surface roughness, hydrophilicity, and microbial accessibility. FTIR spectra supported this compositional modification through the marked reduction in absorbance intensities near 1730 cm⁻^1^ (C=O stretching of hemicellulose) and 1240 cm⁻^1^ (C–O–C stretching of lignin). These molecular-level alterations correlated strongly with the observed increase in weight loss and CO_2_ mineralization, confirming accelerated biodegradation kinetics. The Lanette wax-based (LW) composite demonstrated the highest disintegration and mineralization rates, followed by L, PW, and P, in descending order, consistent with their C/N ratios, where lower C/N values favored faster decomposition.

Although the pots did not achieve the 90% disintegration threshold stipulated by ISO standards within the tested period, the results indicate a clear potential for optimization through compositional refinement and bioactive additive incorporation. The study’s dual analytical framework, coupling kinetic modeling with PCA interpretation, advances the predictive understanding of biocomposite biodegradation, enabling the design of tailored formulations for specific agricultural lifespans. Practically, these biodegradable cultivating pots are promising for short-cycle crops and sustainable farming systems, offering a viable pathway to reduce plastic waste accumulation and enhance soil health.

Future research should focus on integrating bio-catalytic fillers, microbial enhancers, or nanocellulosic reinforcements to accelerate mineralization and meet regulatory standards for full compostability. Moreover, complementing SEM–EDX surface analysis with CHNS and TOC–TN bulk characterization will allow a more rigorous quantification of elemental transformations, bridging microstructural evidence with global biodegradation metrics. The findings collectively underscore the scientific novelty and environmental relevance of this work, contributing a scalable approach toward the development of high-performance, eco-efficient biocomposite pots aligned with global sustainability objectives.

## Data Availability

The datasets used or analyzed during the current study are available from the corresponding author upon reasonable request.
